# Teachings from the French database of TTR familial amyloidotic polyneuropathy (TTR-FAP): large genetic and phenotypic heterogeneity, usefulness of TTR gene testing.

**DOI:** 10.1186/1750-1172-10-S1-P27

**Published:** 2015-11-02

**Authors:** David Adams, Cecile Cauquil, Clovis Adam, Celie Labeyrie, Guillemette Beaudonnet, Anne Mantel, Marie Theaudin

**Affiliations:** 1 Chu Bicetre, Neurology (NNERF), 94270, Le Kremlin Bicêtre, France; 2 Chu Bicetre, Pathology, 94270, Le Kremlin Bicêtre, France; 3 Chu Bicetre, Molecular Biology, 94270, Le Kremlin Bicetre, France; 4 Chu Bicetre, Neurophysiology, 94270, Le Kremlin Bicêtre, France; 5 French Network For Fap, 94270, Le Kremlin Bicetre, France

## Introduction

TTR-FAP is progressive, disabling, irreversible and life-threatening neuropathy due to a point mutation of TTR gene with autosomal dominant transmission. France is a non endemic European country. To study the impact of labeling French reference center for FAP (NNERF) and building of a national network CORNAMYL.

## Methods

In 1986-2014 period, 482 FAP patients were registered in NNERF's database. All carried amyloidogenic TTR gene mutations and Congo positive amyloid deposit (CPAD). To report genotypic and phenotypic varieties of FAP in France in 2008-2014 period and the sensitivity of the tools for diagnosis.

## Results

In 2008-2014 period: 180 new TTR-FAP cases were identified, in 14 additionnal geographical departments (total 81/100), with 9 further TTR gene mutations (total 41).

Mean age was 60 (22-89), a late onset (> or = 50 y) in 69%. Sex ratio: 2.16. Positive family history of FAP 55%. Portuguese origin 18.3%. Diagnosis of FAP was delayed by 2.93y (0.2-13.5) after first symptoms; 69% had walking difficulties including 39% requiring aid.

Five phenotypes were identified: Small Fiber Polyneuropathy (PNP) (43%), All-Fiber SensoryMotor-PNP (25%), Upper Limbs neuropathy (NP) (17%), Ataxic NP (14%), Motor NP (2%). CPAD after nerve biopsy in 19/26pts (73%), Labial Salivary Gland Biopsy (LSGB) in 91/128 pts (71%); 76% required multiple biopsies.

## Conclusions

The French network for TTR-FAP allows to identify new TTR-FAP cases in most of geographical departments with varied phenotypes. The larger use of TTR gene analysis in idiopathic aggressive polyneuropathy cases will help to accelerate diagnosis of TTR-FAP.

